# Complete Coding Genome Sequence of Infectious Hypodermal and Hematopoietic Necrosis Virus Isolated from *Penaeus* (*Litopenaeus*) *vannamei* Shrimp in Ecuador

**DOI:** 10.1128/mra.01143-21

**Published:** 2022-03-17

**Authors:** Leandro Bajaña, Irma Betancourt, Bonny Bayot

**Affiliations:** a Escuela Superior Politécnica del Litoral, Centro Nacional de Acuicultura e Investigaciones Marinas, Guayaquil, Ecuador; b Escuela Superior Politécnica del Litoral, Facultad de Ciencias de la Vida, Guayaquil, Ecuador; c Escuela Superior Politécnica del Litoral, Facultad de Ingeniería Marítima y Ciencias del Mar, Guayaquil, Ecuador; Portland State University

## Abstract

Infectious hypodermal and hematopoietic necrosis virus (IHHNV), recognized as Penaeus stylirostris penstyldensovirus 1 (PstDV1), has been associated with the runt-deformity syndrome (RDS) in cultured Penaeus (Litopenaeus) vannamei and Penaeus monodon shrimp. Here, we report the second published genome sequence of IHHNV, which was isolated from P. vannamei shrimp cultured in Ecuador.

## ANNOUNCEMENT

Whiteleg Penaeus (Litopenaeus) vannamei shrimp is one of the top 10 species in global aquaculture (1), but it is affected by diseases, which impact the economy of the producer countries (2). Infectious hypodermal and hematopoietic necrosis virus (IHHNV) is the smallest of the penaeid shrimp viruses and belongs to the genus *Penstyldensovirus*, subfamily *Densovirinae*, and family *Parvoviridae* (3, 4). The viral genome consists of three open reading frames (ORFs) (5). IHHNV causes high mortality rates in Penaeus stylirostris, but it produces runt-deformity syndrome (RDS) in P. vannamei and Penaeus monodon, which impacts the commercial value at harvest (6–8). On the other hand, insertion of noninfectious IHHNV sequences has been found in the P. monodon genome, which could generate false-positive results for the diagnosis of shrimp infection with IHHNV (9).

Currently, there is only one published genome of IHHNV isolated from cultured shrimp from Ecuador (GenBank accession no. AY362548). This study reports the complete coding genome sequence of a second IHHNV strain (IH19), which was isolated from pleopods of a cultured P. vannamei broodstock sampled in September 2019 in Santa Elena, Ecuador. The genetic material was extracted from macerated pleopods using the phenol-chloroform-isoamyl technique (10). Viral diagnosis was performed by PCR using the IHHNV309F and IHHNV309R primers (11), which are recommended by the World Organization for Animal Health (4) to specifically detect infectious IHHNV forms and exclude the noninfectious related sequences.

**FIG 1:**
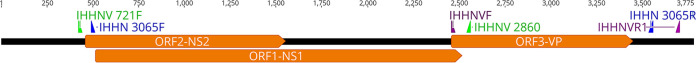
Locations of the different primers in the Ecuadorian IHHNV genome (accession no. AY362548).

The ORFs of IHHNV (NS1, NS2, and VP) were amplified by PCR with three primer pairs (Fig. 1), i.e., IHHN3065F/IHHN3065R (12), IHHNV721F/IHHNV2860R, and IHHNVF/IHHNVR1 (13), using Dr. MAX DNA polymerase (Doctor Protein Inc., South Korea). PCR products were purified using MSNU030 plates (Millipore SAS, Molsheim, France). Sanger sequencing (forward and reverse) was performed using the PCR primers with the BigDye terminator v3.1 sequencing kit and a 3730XL capillary sequencer (Applied Biosystems, Foster City, CA, USA) at Macrogen (Seoul, South Korea). Sequences were edited and aligned (only nucleotides with Phred scores of >20 were used) with Clustal Omega v1.2.2 (14) in Geneious Prime v2020.2.2 (Biomatters, Inc.) using the pairwise alignment tool with default parameters. The consensus sequence of 3,203 bp contained the three ORFs of IHHNV, with 99.4% alignment identity, a GC content of 42.7%, and 84.8% alignment coverage at the nucleotide level, compared with the first IHHNV genome isolated from Ecuador (GenBank accession no. AY362548). Based on comparisons with the available IHHNV reference sequences, this genome was deemed to be coding complete.

This study reports the second genome of an IHHNV strain from Ecuador, compared to the first genome (reported in 2003), which will be relevant for further investigations related to diagnosis improvement and epidemiological studies of evolution and virus pathogenicity, as observations of runts and deformities associated with IHHNV have diminished in cultured shrimp in Ecuador, according to anecdotal information from shrimp farmers.

### Data availability.

The assembled genomic sequences were deposited in GenBank under accession no. OL598344.2.
